# Efficacy of hormone pre‐treatment before ART to improve reproductive outcomes in infertile women with endometriosis: Network meta‐analysis of randomized controlled trials

**DOI:** 10.1002/ijgo.70134

**Published:** 2025-04-12

**Authors:** Gaetano Riemma, Luigi Cobellis, Antonio Simone Laganà, Andrea Etrusco, Luigi Della Corte, Marco Torella, Maria Giovanna Vastarella, Raffaela Maria Carotenuto, Pasquale De Franciscis

**Affiliations:** ^1^ Department of Woman, Child, and General and Specialized Surgery University of Campania “Luigi Vanvitelli” Naples Italy; ^2^ Unit of Obstetrics and Gynecology, “Paolo Giaccone” Hospital, Department of Health Promotion, Mother and Child Care, Internal Medicine and Medical Specialties (PROMISE) University of Palermo Palermo Italy; ^3^ Department of Neuroscience, Reproductive Sciences and Dentistry, School of Medicine University of Naples Federico II Naples Italy

**Keywords:** endometriosis, hormone treatment, in vitro fertilization, live birth rate, pregnancy rate

## Abstract

**Background:**

Hormone pre‐treatment is still used before assisted reproductive technique (ART) in endometriotic women, but evidence supporting this recommendation is conflicting.

**Objectives:**

To evaluate whether hormone pre‐treatment with gonadotropin‐releasing hormone (GnRH) agonists or progestogens could improve fertility in women with endometriosis undergoing ART.

**Search Strategy:**

MEDLINE, LILACS, EMBASE, Scielo.br, PROSPERO, Cochrane at the CENTRAL Register of Controlled Trials, conference abstracts, and international controlled trials registries were searched without temporal, geographic, and language limitations.

**Selection Criteria:**

Randomized controlled trials that enrolled infertile women with endometriosis undergoing in vitro fertilization/intracytoplasmic sperm injection after the application of a stimulation protocol with hormone pre‐treatment were selected and included.

**Data Collection and Analysis:**

We conducted a network meta‐analysis based on the random‐effects model for mixed multiple treatment comparisons to rank the available hormone pre‐treatment by the surface under the cumulative ranking curve area (SUCRA) following the Preferred Reporting Items for Systematic reviews and Meta‐Analyses extension statement for network meta‐analyses. Quality assessment was carried out using the criteria outlined in the Cochrane Handbook for Systematic Reviews of Interventions. Egger test and funnel plot analysis were used for publication bias assessment. The primary outcome was clinical pregnancy rate (CPR). Secondary outcomes were live birth rate (LBR), pregnancy loss rate (PLR), and implantation rate (IR).

**Main Results:**

Nine studies with 2087 women were included. Ultralong (3 months GnRH agonist) (SUCRA 24.5%) and long protocols (1 month GnRH agonist) (SUCRA 24.9%) as well as progestins (SUCRA 28.8%) showed similar results to no treatment (SUCRA 21.8%) in terms of post‐ART CPR. Regarding the LBR, no treatment (SUCRA 50.0%) showed highest rates relative to progestins (SUCRA 7.0%), and long (SUCRA 36.6%) and ultralong (SUCRA 6.4%) protocols. For PLR, no treatment (SUCRA 57.9%), followed by long protocol (SUCRA 18.4%), ultralong protocol (SUCRA 12.3%), and progestins (SUCRA 11.4%) showed the greatest degree of reduction. The long (SUCRA 45.0%) and ultralong (SUCRA 39.5%) protocols seemed more effective in increasing IR than did than progestins (SUCRA 15.5%).

**Conclusions:**

The increased number of implanted pregnancies using a GnRH agonist protocol does not lead to higher clinical pregnancies or live births. Currently, there is no indication for hormone pre‐treatment before ART in women with endometriosis as it does not increase fertility chances.

## 1 INTRODUCTION

### 1.1 Background

Endometriosis is defined by the development of tissues that resemble endometrial tissue outside the uterine cavity.^1^

It affects around 10% of women of reproductive age and 20%–50% of infertile women.^2^ It is a chronic, estrogen‐dependent inflammatory condition. A high number of assisted reproductive technology (ART) procedures are performed in endometriosis patients to increase pregnancy rates.^3^ In vitro fertilization (IVF), rather than any surgical intervention, is a crucial approach to improve reproductive outcomes of infertile women with endometriosis.^4^ Although it is known that endometriosis can impair the ability to conceive due to certain factors (e.g. formation of tubal adhesions), it is still unclear how exactly endometriosis impairs fertility through pathophysiological processes.^5^

Continuous gonadotropin‐releasing hormone (GnRH) agonist causes pituitary suppression and reduces ovarian steroidogenesis, which deprives existing endometriotic lesions of their primary growth factor.^6^ By using different mechanisms, these medications also directly affect the proliferation of endometrial cells.^7^ Therefore, pre‐treatment with a GnRH agonist might restore the hostile, inflammatory peritoneal environment caused by endometriosis as well as other negative effects, including poor folliculogenesis, which reduces the quality of oocytes and impairs endometrial receptivity. After in vitro fertilization (IVF), these effects could lead to better clinical results.^8^, ^9^

In addition to having an inhibitory impact on cytokines like interleukin‐8 (IL‐8), progestins, including dienogest and medroxyprogesterone acetate (MPA), have been reported to have a higher cytoreductive effect on endometriosis lesions than GnRH agonist.^10^ As a result, some studies hypothesized that progestins before IVF and embryo transfer (ET) may enhance the procedure's success rates for implantation and pregnancy.^11^, ^12^

Over the last 20 years, several studies have been conducted to evaluate the efficacy of different strategies in boosting the reproductive outcomes of women with endometriosis undergoing IVF/intracytoplasmic sperm injection (ICSI). A variety of approaches, including pre‐treatment with progestogens and GnRH agonists for one or more months, were compared among each other or with a placebo.^13^, ^14^, ^15^ However, as the available evidence is still limited and conflicting, there is still a great deal of uncertainty regarding which hormone treatment before ART (long [1 month GnRH agonist pre‐treatment], ultralong [3 months GnRH agonist pre‐treatment] or other hormone treatment) will effectively increase pregnancy chances and, of these, which protocol is more effective than the others.

### 1.2 Objectives

The aim of this systematic review and network meta‐analysis is to summarize the current evidence on the usefulness of hormone treatment before ART in patients with endometriosis and the effect of this pre‐treatment on subsequent fertility outcomes using direct or indirect comparisons between the available strategies.

## 2 METHODS

This network meta‐analysis was carried out according to the principles of the Cochrane Handbook for Systematic Reviews of Interventions^16^ and the procedures specified by Mbuagbaw et al.^17^ It adhered to the Preferred Reporting Items for Systematic reviews and Meta‐Analyses (PRISMA) extension statement for network meta‐analyses (PRISMA‐NMA).^18^ The study protocol was registered in the International Prospective Register of Systemiatic Reviews (PROSPERO) database (CRD42023446300) on July 28, 2023.

### 2.1 Information sources and search strategy

Electronic databases including MEDLINE (accessed through PubMed), LILACS, EMBASE, Scielo.br, and PROSPERO were searched with the following keywords and Medical Subject Heading (MeSH) terms: “ovarian stimulation” or “assisted reproduction technique” or “in vitro fertilization” and “endometriosis” without any date restriction. The search string was modified according to each database's format (Table S1).

The search results were filtered to show only randomized controlled trials (RCTs). Additionally, searches on CINAHL, PsycINFO, and AMED were performed to look for additional pertinent publications to lessen the publication bias. We also searched Clinicaltrials.gov, Cochrane Central Register of Controlled studies, and the WHO International Clinical Trials Registry Platform (ICTRP) to find more RCTs. In addition, the gray literature (NTIS, PsycEXTRA) was reviewed to look for conference abstracts at both international and domestic levels. To find additional publications that were missed during the first search, we additionally looked through the references of the included research and the associated reviews.

There were no restrictions based on geography or language. The search did not include commentary, letters to the editor, editorials, or second opinions.

### 2.2 Study selection

The inclusion criteria considered any RCT that enrolled infertile women with endometriosis, or previously treated for endometriosis, undergoing ART after the application of hormone pre‐treatment before ovarian stimulation. Endometriosis was diagnosed laparoscopically and/or with ultrasonography/magnetic resonance imaging as deep, superficial, or ovarian, according to the revised criteria of the American Society for Reproductive Medicine (rASRM) classification system .^19^ The score was assigned by the authors of the original articles. The exclusion criteria were: quasi‐randomized trials and trials without randomization and studies including patients undergoing intrauterine insemination or ovulation induction without IVF/ICSI attempts.

### 2.3 Data extraction

The abstraction forms were created specifically for this network meta‐analysis. The following important facts were noted: patient descriptions, study duration, setting, endometriosis stage, treatment types, outcomes assessed, mean follow‐up time, results, and quality characteristics.

Two authors (GR and LC) independently examined and categorized each abstract. The same two writers conducted a comprehensive text analysis of the chosen studies and separately collected relevant data regarding the research features and the results of interest to reach agreement on potential relevance. The reviewers discussed each inconsistency, and after consulting a third author (PDF), agreement was achieved. When the research methods indicated that other outcome data were gathered, unpublished data were obtained, if necessary, by contacting the authors of the original studies directly.

### 2.4 Assessment of risk of bias

The risk of bias in each of the included studies was evaluated using the criteria outlined in the *Cochrane Handbook for Systematic Reviews of Interventions*.^16^ Each included trial's critical investigation focused on the following seven domains because it was clear that these problems contributed to inaccurate estimates of the effects of treatments: (1) random sequence generation; (2) allocation concealment; (3) blinding of participants and staff; (4) blinding of outcome assessment; (5) incomplete outcome data; (6) selective reporting; and (7) other bias. It was determined if there was a “low risk,” “high risk,” or “unclear risk” of bias in the writers' assessments.

Three authors (MT, RMC and MGV) independently rated the risk of bias assessment. Conflicts were settled by discussion with a fourth reviewer (PDF).

### 2.5 Primary and secondary outcomes

The primary outcome of this network meta‐analysis was the clinical pregnancy rate (CPR), defined for the included studies as an ultrasonographic visualization of one or more intrauterine gestational sacs with fetal heart activity. The secondary outcomes were live birth rate (LBR), defined as the birth of a living fetus after 24 weeks of gestational age; implantation rate, defined as the number of gestational sacs divided by the number of embryos transferred; and pregnancy loss rate (PLR), defined as a spontaneous interruption of pregnancy before 12 weeks of gestation.

### 2.6 Data synthesis

STATA version 14.1 (StataCorp, College Station, TX, USA) was used for all data analysis and graphical representations. The command <network meta consistency> was used to statistically verify the network assumption of overall consistency for each result of interest. The Separating Indirect from Direct Evidence (SIDE)‐splitting approach, utilizing the command <network meta inconsistency>, was then employed for the local test on loop inconsistencies. The consistency assumption was accepted when it was discovered that there was no discrepancy in both the global and local tests. In this instance, the consistency model showed that both direct and indirect comparisons in this study were certain to provide significant findings, and that any discrepancies in the results were only from the intervention's effects and random mistakes.

Using the Der Simonian and Laird random‐effects model, the summary measures were reported as relative risks (RRs) or odds ratios (ORs) for categorical variables and mean differences (MDs) for continuous variables, with 95% confidence intervals (CIs). A Higgins *I* ^2^ score greater than 0% was utilized to pinpoint possible heterogeneity. Sensitivity analyses were carried out in situations of considerable heterogeneity to identify the pertinent sources of heterogeneity.

The funnel plot's symmetry was examined to determine any potential publication bias. The Egger test was employed to quantify the publication bias, however, as different observers may have come to different results from the same funnel plot. To analyze the efficacy of the various hormone regimens and to rank the treatments to determine superiority, a ranking plot (surface under the cumulative ranking curve area [SUCRA]) and a prediction interval plot were developed for each examined outcome.

## 3 RESULTS

### 3.1 Study selection

**FIGURE 1:**
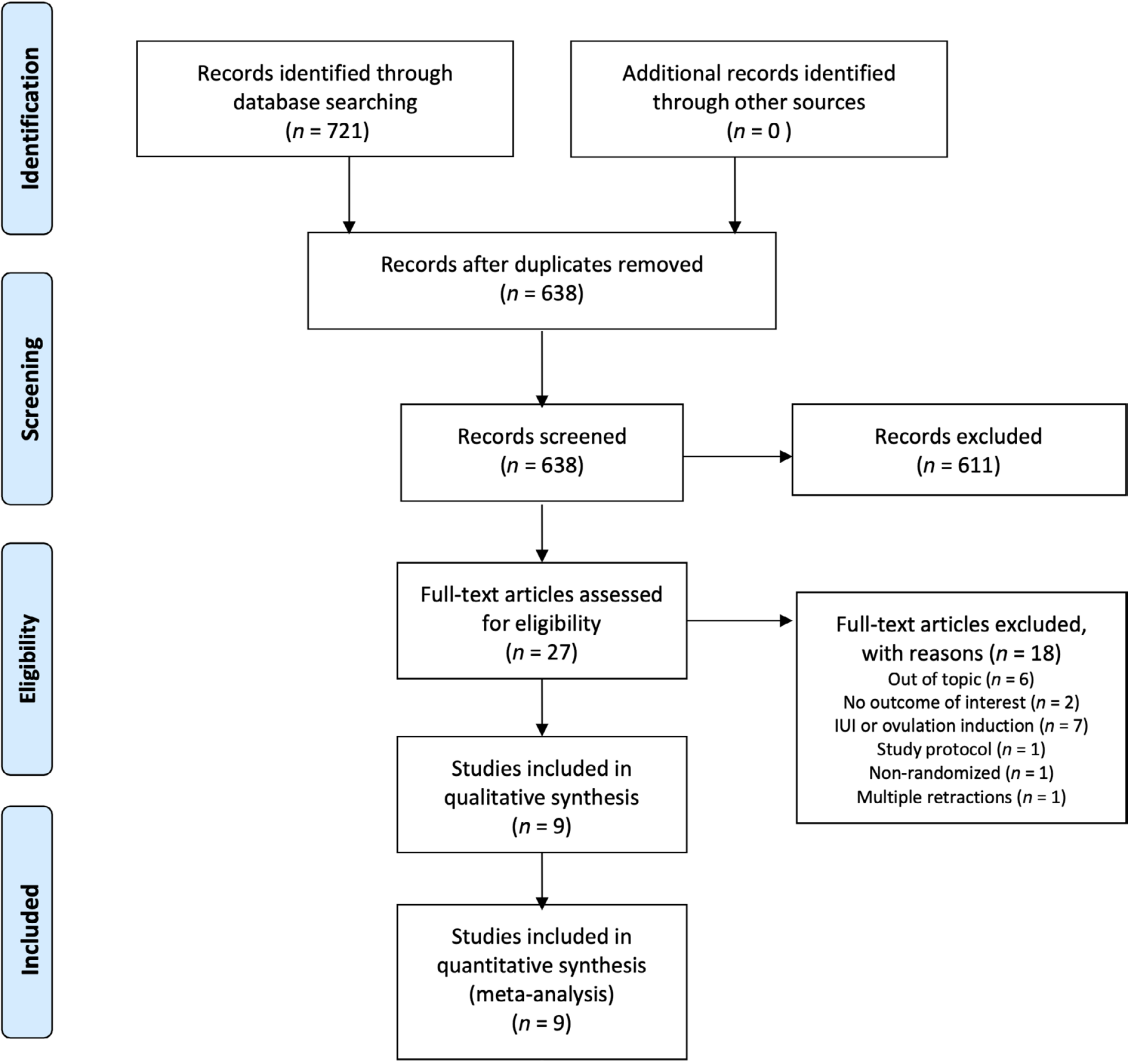
Preferred Reporting Items for Systematic reviews and Meta‐Analyses flowchart of included studies in systematic review and network meta‐analysis.

In all, 721 studies were initially identified using database searches. Of those, 83 were removed as duplicates. After title and abstract screening, 611 papers were subsequently removed (Figure 1). Twenty‐seven studies underwent full text assessment, of which one was removed for being non‐randomized, two for not including outcomes of interest, seven for considering intrauterine insemination or ovulation induction rather than IVF/ICSI, and one for being a study protocol only (Figure 1). Another study was excluded before final analysis due to multiple retractions for the first author of the paper.

Nine studies,^14^, ^20^, ^21^, ^22^, ^23^, ^24^, ^25^, ^26^, ^27^ including 2087 infertile women with endometriosis, were included in the systematic review and network meta‐analysis (Figure 1).

### 3.2 Study characteristics

The studies were carried out between 2002 and 2022. Women with mild, moderate, or severe endometriosis (stages I–IV according to rASRM criteria) were included (Table 1).

**TABLE 1:** Characteristic of studies qualified for systematic review and network meta‐analysis.

Study	Year	Design	Period	Location	Population	Endometriosis stage	Intervention 1	Intervention 2	Sample size	Embryo transfer type	Outcomes
Guo et al.	2022	Single‐center RCT single blind	March 2017 to September 2017	China	Patients undergoing IVF after treatment for endometriosis	Severe (rASRM III–IV) or Endometrioma >3 cm	MPA (10 mg/day) from third day of menstrual cycle onward	1‐month long protocol with triptorelin depot 3.75 mg protocol on the second day of menstrual cycle	300	Fresh or frozen	CPR, LBR, PLR, IR
Tomassetti et al.	2021	Single‐center RCT no‐blind	June 2013 to December 2016	Belgium	Women with a history of therapeutic surgery for endometriosis and an indication for ART	NA	3‐month ultralong (triptorelin depot 3.75 mg) protocol	1‐moth long agonist protocol (triptorelin 0.1 mg/day)	42	Fresh	CPR, LBR, PLR
Khalifa et al.	2021	Single‐center RCT single blind	August 2018 to October 2019	Egypt	Women having a confirmed diagnosis of endometriosis by laparoscopy	NA	3‐month ultralong protocol (leuprorelin depot 3.75 mg on monthly basis)	Dienogest 2 mg orally daily for 3 month	134	Fresh	CPR, PLR
Rodriguez‐Tarrega et al.	2020	Single‐center RCT single blind	March 2012 to March 2015	Spain	Women with a laparoscopic diagnosis of endometriosis	rASRM I–IV	3‐month ultralong protocol (triptorelin depot 3.75 mg intramuscularly on monthly basis)	Placebo (3 injections of saline with the same disposal and the same monthly basis)	100	Fresh or frozen	CPR, LBR, PLR
Kaponis et al.	2019	Multicentric RCT unclear blinding	May 2004 to December 2018	Greece	Women with laparoscopically documented endometriosis	Mild (rASRM I–II)	3‐month ultralong protocol (leuprorelin depot 3.75 mg every 28 day)	No treatment	400	Fresh	CPR
Tamura et al.	2019	Single‐center RCT single‐blind	February 2011 to November 2015	Japan	Women with endometriosis or endometrioma (<4 cm) diagnosed by ultrasonography o MRI, laparoscopy	Severe (rASRM III or IV) or ovarian endometriosis (cyst <4 cm)	Dienogest 2 mg (administered orally every day for 12 week from 3 month prior to the IVF‐ET cycle)	Long protocol (900 μg/day buserelin acetate, administered from the mid‐luteal phase in the previous cycle)	68	Fresh or frozen	CPR, LBR, PLR, IR
Decleer et al.	2016	Single‐center RCT single blind	February 2012 to March 2014	Belgium	Women with a history of therapeutic surgery for endometriosis and an indication for treatment with ART	rASRM I or II	3‐month ultralong goserelin 3.6 mg protocol (the first being given on the second day of the cycle)	Long protocol (nasal 900 buserelin acetate μg/day)	120	Fresh or frozen	CPR
Rickes et al.	2002	Single‐center RCT no blind	May, 1999 to May, 2001	Germany	Women with history of surgery for endometriosis and indication for ART	rASRM II–IV	3‐month ultralong goserelin 3.6 mg protocol (the first being given on the second day of the cycle)	Long agonist protocol (GnRH agonist NA)	110	Fresh	CPR
Surrey et al.	2002	Single‐center RCT unclear blinding	NA	USA	Women surgically treated for endometriosis and an undergoing ART	rASRM I–IV	3‐month ultralong protocol (leuprorelin acetate depot 3.75 mg intramuscular. every 28 day for three injections)	No treatment	51	Fresh	CPR

Abbreviations: ART, assisted reproduction technique; CPR, clinical pregnancy rate; GnRH, gonadotropin‐releasing hormone; IM, intramuscular; IR, implantation rate; LBR, live birth rate; MPA, medroxyprogesterone acetate; NA, not available; PLR, pregnancy loss rate; rASRM, revised American Society for Reproductive Medicine; RCT, randomized controlled trial.

Studies were conducted in Belgium,^20^, ^25^ Egypt,^21^ Spain,^22^ Greece,^23^ Germany,^26^ the United States,^27^ Japan^24^ and China.^14^ Sample sizes ranged from 42^20^ to 300^14^ (Table 1).

The inclusion and exclusion criteria provided by the studies examined in the network meta‐analysis are listed in Table 2.

**TABLE 2:** Inclusion and exclusion criteria of included papers.

Study	Inclusion criteria	Exclusion criteria
Guo et al.	Surgical confirmation of severe ovarian endometriosis, stage III–IV endometriosis according to rASRM; age ≤ 40 year; regular menstrual cycle (25–35 day per cycle) in the prior 3 month; antral follicle count more than 4 and less than 20 on menstrual cycle day 2–3; basal serum FSH ≤10 IU/L	Documented ovarian failure, including basal FSH >10 IU/L or no antral follicles on ultrasound; PCOS; hydrosalpinx; adenomyosis on the laparoscopy or laparotomy; hormone treatment within 3 month; mild or peritoneal endometriosis; moderate to severe intrauterine adhesion
Tomassetti et al.	History of therapeutic surgery for endometriosis and an indication for treatment with ART; age 18–39 year; EFI available or calculable, normal uterine cavity; basal FSH on days 2–5 lower than 20 IU/L	Presence of large intramural fibroids (>3 cm) without impression on the cavity previous ART treatment with low response (<4 mature oocytes obtained), non‐obstructive azoospermia
Khalifa et al.	Confirmed diagnosis of endometriosis by laparoscopy in the last 2 years, age <40 years, body mass index <35 kg/m^2^	Already treated with GnRH agonist; liver or kidney disease, diminished ovarian reserve (e.g., high FSH level >12 IU/L, low AMH level <1.1 ng/mL or low antral follicle number <7)
Rodriguez‐Tarrega et al.	Patients <40 year old; laparoscopic diagnosis and surgical resection of endometriosis or ovarian endometriotic cysts; BMI <28; infertility with indication for IVF or ICSI	FSH measured in early follicular phase >12 IU/L; major disorders
Kaponis et al.	Infertile women (after >1 year of attempts), aged 29–38 year, laparoscopically documented endometriosis	Sonographic evidence of ovarian endometrioma >2 cm in mean diameter, with early follicular phase FSH levels >12 mIU/mL; severe male factor infertility
Tamura et al.	Infertile women 20–40 year of age with endometrioma (<4 cm) or endometriosis diagnosed by ultrasonography or MRI or laparoscopy. Classification via laparoscopy	Use of hormonal contraceptives or other hormonal therapies or who had a disease condition that might interfere
Decleer et al.	Women with endometriosis younger than 38 year with indication for IVF	Patients >38 year old, severe male factor infertility, deep fibrosing endometriosis of the rectovaginal septum and uterine pathology, major endocrine diseases
Rickes et al.	NA	Lack of desire to conceive, age >40 year, severe male factor
Surrey et al.	Infertile patients with endometriosis documented at laparoscopy or laparotomy; regular menstrual cycle (every 26–33 day) and candidates for autologous IVF‐ET	Early follicular phase serum FSH levels >12 mIU/mL and evidence of ovarian endometrioma

Abbreviations: AMH, anti‐Müllerian hormone; EFI, endometriosis fertility index; ET, embryo transfer; FSH, follicle‐stimulating hormone; ICSI, intracytoplasmic sperm injection; IVF, in vitro fertilization; NA, not available; PCOS, polycystic ovary syndrome; rASRM, revised American Society for Reproductive Medicine.

Five studies^20^, ^21^, ^23^, ^26^, ^27^ analyzed fresh ETs only, while four^14^, ^22^, ^24^, ^25^ used both fresh and frozen ETs.

The types of hormone pre‐treatments used were long (1 month) or ultralong (3 months) treatments with GnRH agonist, and 1 month of progestin‐only treatment (dienogest or MPA) (Table 1). No treatment or placebo was assumed as reference for direct and indirect comparisons.

For the ultralong GnRH agonist, two studies used goserelin 3.6 mg,^25^, ^26^ two studies employed triptorelin depot 3.75 mg, while one study used leuprorelin depot 3.75 mg.^21^

Concerning the long GnRH agonist pre‐treatment, two studies^14^, ^20^ utilized triptorelin depot 3.75 mg, while two researches^24^, ^25^ used 900 μg/day of nasal buserelin acetate. Dienogest was used in two studies^21^, ^24^ while Guo et al. employed MPA 10 mg/day^14^ (Table 1).

In all included studies, the treatment and control groups were comparable in terms of age, total number of oocytes, and number and quality of transferred embryos.

#### 3.2.1 Risk of bias of included studies

In Figure S1a, the quality of the methodology employed for each trial is shown, and in Figure S1b, a summary of the methodology's quality across all trials is shown in percentages. Most of the studies that were included in the analysis had a low bias risk. However, two out of nine trials^20^, ^26^ were considered high risk for blinding‐related scores due to the absence of blinding of personnel and participants, while two studies^23^, ^27^ reported no blinding or no information about blinding; therefore, such studies were considered to have unclear risk (Figure S1). Prior to the enrollment of participants, all included trials were recorded in valid prospective registries.

### 3.3 Synthesis of results

#### 3.3.1 Clinical pregnancy rate

**FIGURE 2:**
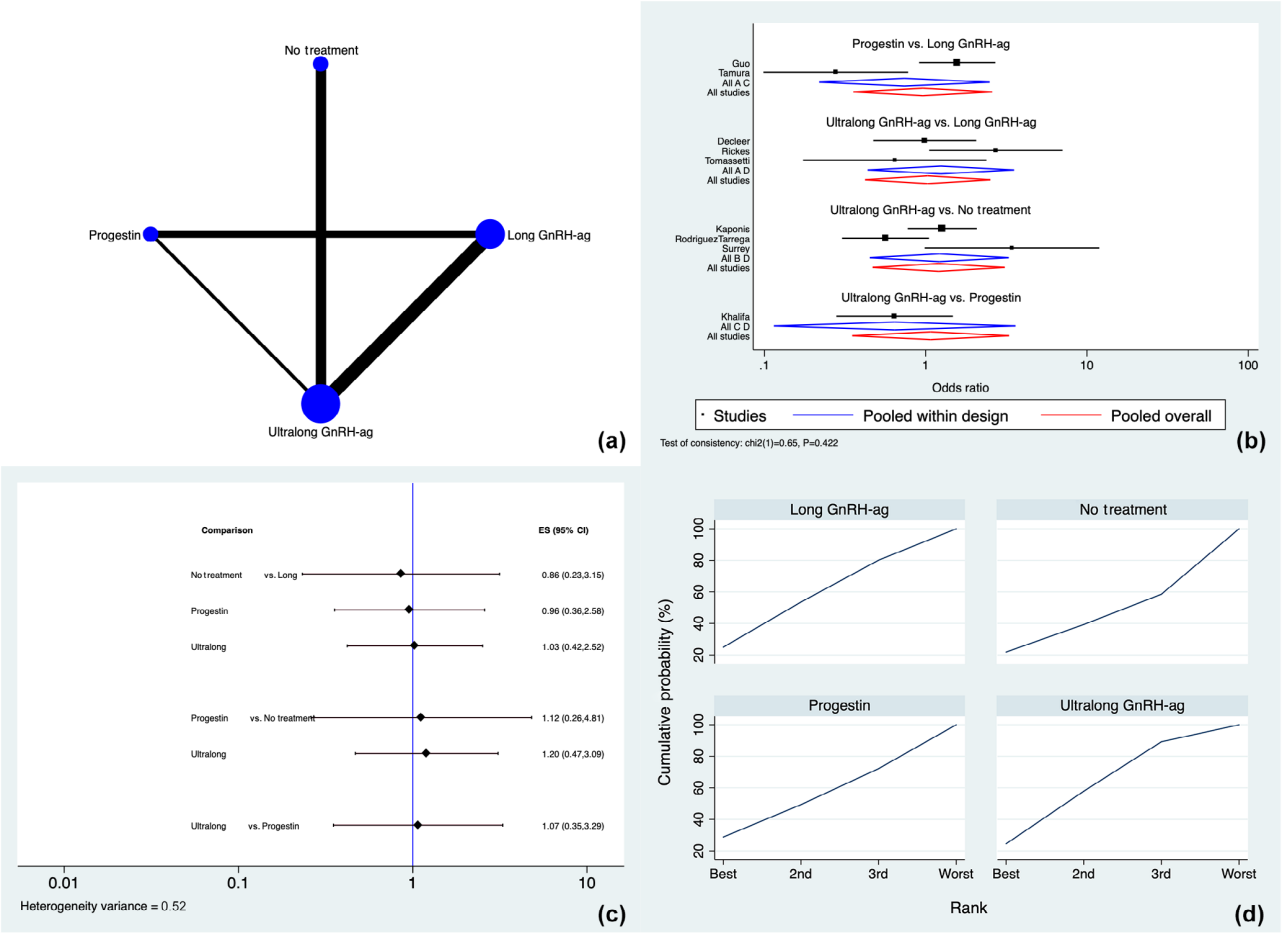
Clinical pregnancy rate. (a) Network of comparisons of interventions analyzed in included studies. (b) Forest plot for the outcome. (c) Prediction interval plot. (d) Ranking plot according to surface under the cumulative ranking curve area analysis. GnRH‐ag, gonadotropin‐releasing hormone agonist.

All nine studies^14^, ^20^, ^21^, ^22^, ^23^, ^24^, ^25^, ^26^, ^27^ analyzed the CPR. Long and ultralong GnRH agonist treatment, progestins and no treatment were used as hormone pre‐treatment protocols. Figure 2a shows the frequency of studied therapies and most accurate direct comparisons. The inconsistency analysis showed that global inconsistency was not present (*P* = 0.422).

The SIDE analysis showed that there were no differences between the consistency and inconsistency models or between the direct and indirect estimates in the closed loops considered for the network (local inconsistency) (Table S2).

The symmetrical funnel plot (Figure S2) and Egger's test (*P* = 0.669) for this primary outcome showed no evidence of substantial publication bias in this network meta‐analysis.

Figure 2b,c show the forest plot and predictive interval plot, respectively, which illustrate the effect of various techniques on the increase of CPR. According to the inspection of the forest and prediction interval plots, no discernible differences between the treatments were found.

**FIGURE 3:**
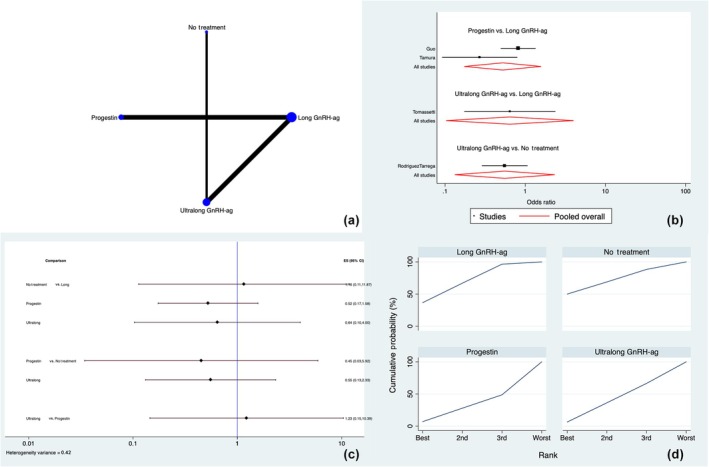
Live birth rate. (a) Network of comparisons of interventions analyzed in included studies. (b) Forest plot for the outcome. (c) Prediction interval plot. (d) Ranking plot according to surface under the cumulative ranking curve area analysis. CI, confidence interval; GnRH‐ag, gonadotropin‐releasing hormone agonist.

According to the SUCRA ranking (Figure 3d), there were no marked differences among the different protocols, with the ultralong GnRH agonists (24.5%), progestins (28.8%), and long GnRH agonists (24.9%) having similar chances of being ranked first as no treatment (21.8%).

#### 3.3.2 Live birth rate

Four studies^14^, ^20^, ^22^, ^24^ calculated the LBR following fresh or frozen ET. Long and ultralong GnRH agonists, progestins, and placebo/no treatment were directly and indirectly compared (Figure 3a). The entire analysis found non‐significant discrepancy (*P* = 0.999). The closed loops tested for this outcome did not exhibit any local inconsistency (Table S2).

The analysis of the network forest plot and prediction interval plot did not show any significant differences among the three treatments (Figure 3b,c). Similarly, the SUCRA ranking showed that no treatment or placebo (50.0%) had more chance of being the best treatment of choice compared with long GnRH agonist (36.6%), progestins (7.0%), and ultralong GnRH agonist (6.4%) (Figure 3d).

#### 3.3.3 Pregnancy loss rate

**FIGURE 4:**
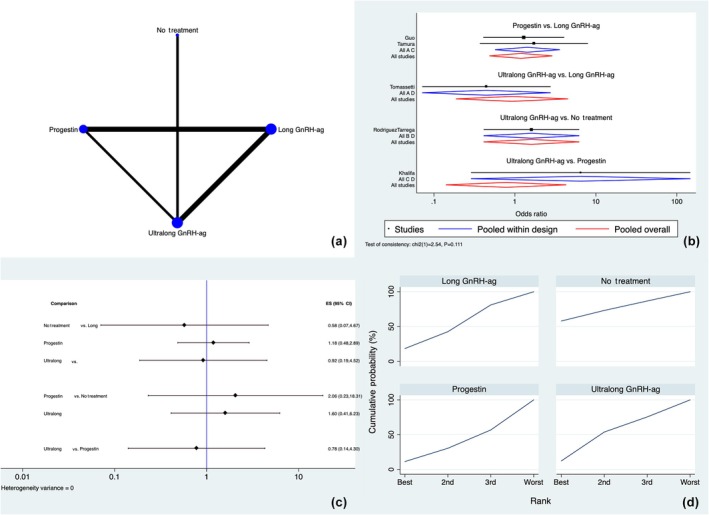
Pregnancy loss rate. (a) Network of comparisons of interventions analyzed in included studies. (b) Forest plot for the outcome. (c) Prediction interval plot. (d) Ranking plot according to surface under the cumulative ranking curve area analysis. CI, confidence interval; GnRH‐ag, gonadotropin‐releasing hormone agonist.

The PLR was evaluated in five out of nine studies.^14^, ^20^, ^21^, ^22^, ^24^ Long and ultralong GnRH agonists, progestins, and placebo/no treatment regimens were the four therapies used among the available studies (Figure 4a). Inconsistency was not found throughout the investigation (*P* = 0.110). There were no local inconsistencies in the closed loops examined for this result (Table S2).

There were no discernible changes between the three treatments according to the study of the network forest plot and prediction interval plot (Figure 4b,c).

According to SUCRA analysis, the absence of pre‐stimulation treatment had an increased chance of being ranked first for fewer pregnancy losses among the evaluated treatments (57.9%), followed by the long protocol (18.4%), the ultralong protocol (12.3%), and progestins (11.4%) (Figure 4d).

#### 3.3.4 Implantation rate

**FIGURE 5:**
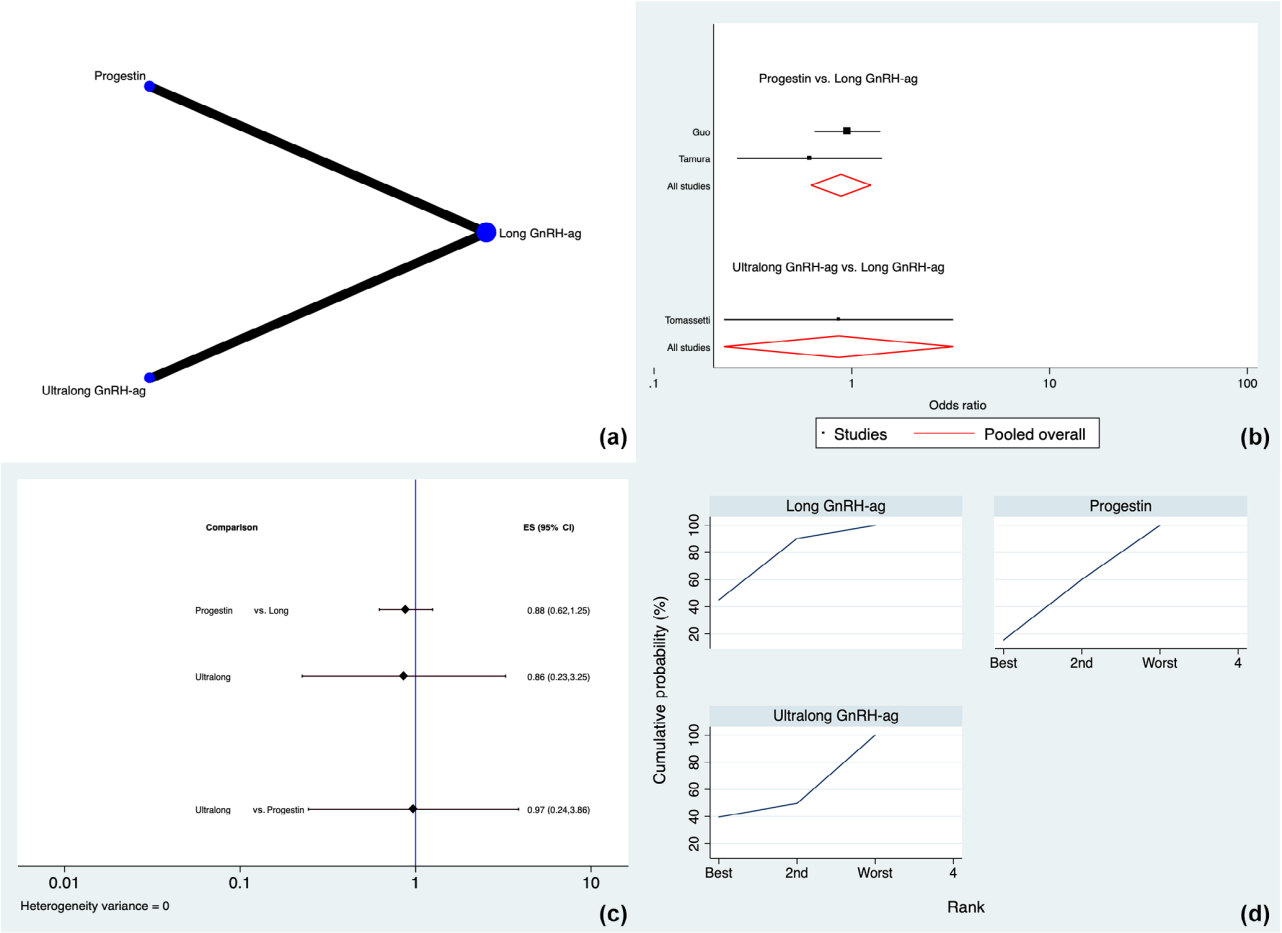
Implantation rate. (a) Network of comparisons of interventions analyzed in included studies. (b) Forest plot for the outcome. (c) Prediction interval plot. (d) Ranking plot according to surface under the cumulative ranking curve area analysis. CI, confidence interval; GnRH‐ag, gonadotropin‐releasing hormone agonist.

Three out of nine studies^14^, ^20^, ^24^ investigated the implantation rate. The three pre‐treatments used in the available research were long GnRH agonists, ultralong GnRH agonists, and progestin‐based protocols (Figure 5a). Throughout the research, no inconsistencies were found (*P* = 0.999). The closed loops tested for this finding showed no local discrepancies (Table S2).

The network forest plot and prediction interval plot analyses revealed no significant differences between the three treatments (Figure 5b,c). Long GnRH agonist pre‐treatment showed higher odds of being classified first for higher post‐ET implantation rates among the examined therapies (45.0%), followed by ultralong protocols (39.5%) and progestins (15.5%) according to SUCRA ranking plots (Figure 5d).

#### 3.3.5 Subgroup analyses

Subgroup analyses on specific subsets (rASRM stage, surgical treatment, and ET type) were carried out to better address between‐study heterogeneity. For each analysis, no source of inconsistency was retrievable (*P* = 0.999).

##### rASRM stage

To evaluate the effects of hormone pre‐treatment on different stages of endometriosis, we performed a subgroup analysis on studies that recruited only women with mild (rASRM I–II, two studies) or severe (rASRM III–IV, two studies) endometriosis.

For severe endometriosis, long GnRH agonist protocols and progestins were directly compared. There were no significant differences between the two approaches for CPR (OR 0.70 [95% CI 0.13–3.81], *P* = 0.68; *I* ^2^ = 88%), LBR (OR 0.52 [95% CI 0.18–1.51], *P* = 0.23; *I* ^2^ = 70%), PLR (OR 1.43 [95% CI 0.57–3.58], *P* = 0.45; *I* ^2^ = 0%) and implantation rate CPR (OR 0.88 [95% CI 0.62–1.25], *P* = 0.48; *I* ^2^ = 0%).

In women with mild endometriosis, long and ultralong GnRH agonists and no treatment were directly and indirectly compared to retrieve the CPR (Figure S3a), while there were no data for the remaining outcomes. The network forest plot and prediction interval plot analyses revealed no significant differences between the three treatments (Figure S3b,c), with SUCRA analysis showing no treatment as the best approach (SUCRA 60.4%) (Figure S3d).

##### Surgical treatment

In seven studies, women were surgically treated for endometriosis before IVF. As no significant differences were notable for the CPR (seven studies^14^, ^20^, ^22^, ^23^, ^25^, ^26^, ^27^) among long and ultralong GnRH agonists, progestins and no treatment (Figure S4a–d), SUCRA analysis showed no agreement for the best approach, with long GnRH agonists (SUCRA 37.2%) and no treatment (SUCRA 36.2%) as the highest ranked. Concerning the LBR (three studies^14^, ^20^, ^22^), no significant differences were noted (Figure S5a–d), with no treatment and long GnRH agonist approaches showing the highest likelihood for the best treatment (SUCRA 53.5% and 37.1%, respectively). PLR, evaluated in three studies,^14^, ^20^, ^22^ did not differ among the approaches (Figure S6a–d), with no treatment having a higher chance of being the best approach (SUCRA 67.3%). Regarding the implantation rate, reported in two studies,^14^, ^20^ as no significant differences were noted, long and ultralong GnRH agonists and progestins were equally likely to be the best treatment (SUCRA 35.5%, 38.3%, and 26.3%, respectively) (Figure S7b–d).

Conversely, women with untreated or unclear treatment status regarding their diagnosis of endometriosis were considered in two studies,^21^, ^24^ directly and indirectly comparing ultralong GnRH agonists, long GnRH agonists, and progestins for CPR and PLR, showing that both progestins (OR 0.28 [95% CI 0.10–0.78]) and ultralong GnRH agonists (OR 0.18 [95% CI 0.05–0.67]) were less efficacious than long GnRH agonists for increasing the CPR (Figure S8a–d), also confirmed best treatment in SUCRA analysis (SUCRA 99.0%). Regarding the PLR, the long GnRH agonist approach was less efficacious than progestins (OR 0.28 [95% CI 0.10–0.78]) and the ultralong approach (OR 0.18 [95% CI 0.05–0.67]), with the latter having the highest likelihood of being the best approach (SUCRA 84.9%) (Figure S9a–d). No data were available for the LBR and implantation rate.

##### ET type

Fresh‐only ETs were involved in five studies, with all five studies^20^, ^21^, ^23^, ^26^, ^27^ reporting the CPR and two studies^20^, ^21^ the PLR, while there were no reports for LBR and implantation rate. Regarding the CPR, forest and interval plots showed that there were no significant differences among long and ultralong GnRH agonists, progestins, and no treatment (Figure S10a–d). SUCRA analysis reported that progestins had increased chances of being the best treatment (SUCRA 65.4%). PLR did not show significant differences among treatments (Figure S11a–d), with an ultralong GnRH agonist approach showing the greatest likelihood of being the best treatment (SUCRA 68.3%). We were unable to assess all the outcomes for frozen ETs as no study reported such approach selectively.

## 4 DISCUSSION

This quantitative synthesis and network meta‐analysis of RCTs showed that, in terms of CPR and LBR, there is no clear advantage in choosing progestins or a long or ultralong GnRH agonist pituitary downregulation protocol to improve fertility before IVF/ICSI in women with ovarian or pelvic endometriosis. Even if the use of a long pituitary downregulation were to give an increased number of implanted pregnancies, such an advantage is not retrievable in terms of higher clinical pregnancies or live birth rates.

### 4.1 Comparison with existing literature

Recent studies have revealed that GnRH‐a medication might significantly decrease inflammatory reactions and angiogenic responses and generate a surprisingly high degree of apoptosis in women with endometriosis, in addition to its hypoestrogenic effects.^28^

The ovarian response to gonadotropins may be suppressed by extremely prolonged pituitary desensitization. Kaponis et al.^23^ found that, although not statistically significant, women who received GnRH agonists for 3 months required more gonadotropin units for ovarian stimulation. Additionally, the women with endometriosis did not have statistically different numbers of follicles or collected oocytes.^23^

Some studies proposed that the effectiveness of reproductive surgery for endometriosis prior to the initiation of ART therapy might account for the absence of treatment benefit in the ultralong suppression group.^5^, ^20^ Only a small percentage of patients were seen in Tomassetti et al.'s study with probable evidence of recurring endometriosis based on reported symptoms and/or imaging in the ultralong group. All patients had recently undergone comprehensive surgical treatment for their endometriosis. Therefore, in this patient population, the benefits of complete surgery before ART may offset or surpass any purported benefits of the ultralong procedure.^20^

Reactive oxygen species generate oxidative stress, which is a key factor in the pathophysiology of endometriosis. Iron and heme are released into the peritoneal environment by an increase in erythrocytes in women with endometriosis. In peritoneal macrophages, increased NF‐kB owing to iron overload causes pro‐inflammatory, growth, and angiogenic factors to be produced in endometriosis patients.^29^ As a result, it was assumed that dienogest or other progestins would have a positive impact on the clinical outcomes of IVF‐ET, as GnRH agonist, by suppressing endometriosis lesions, inflammatory cytokine levels, and oxidative stress.^14^, ^24^ Findings from Tamura et al.^24^ and Guo et al.^14^ indicated that progestin therapy shortly prior to IVF/ET did not enhance reproductive outcomes in infertile women with endometriosis.^14^, ^24^ Infertile women with endometriosis who had therapy with dienogest prior to IVF/ET had fewer developing follicles, retrieved oocytes, fertilized oocytes, and blastocysts, which led to a lower pregnancy rate.^4^, ^14^, ^24^ To fully understand how they affect the oxidative state of the peritoneal cavity in infertile women with endometriosis, more research is required.

### 4.2 Strengths and limitations

This network meta‐analysis had several limitations. First, there were not many studies that met the inclusion criteria, and the sample sizes were modest. Second, as only two studies performed subgroup analyses in accordance with the subtype of therapy, additional outcomes and subgroup analysis were not assessed in this regard owing to data gathered from original research.

Another constraint is the paucity of knowledge regarding the existence of adenomyosis in patients enrolled in the original trials. A pre‐treatment or a lengthy regimen with GnRH agonists may be useful in these individuals, as has been seen in several studies.^30^ Adenomyosis has a negative impact on the clinical results of IVF.^31^ This network meta‐analysis did not evaluate this issue as only one out of the nine included studies evaluated the co‐presence of adenomyosis, without providing separate data.^20^

Additionally, significant clinical variability exists among the included studies. The populations studied varied in terms of endometriosis severity (ranging from rASRM stage I to IV). For this reason, a subgroup analysis on mild (rASRM stage I–II) and severe (stage III–IV) endometriosis was carried out, confirming the lack of effectiveness of hormone pre‐treatment for both subgroups.

For certain outcomes, most of trials came from single centers. Although independent participants were included, there is a greater risk for selection, performance, detection, and reporting biases relative to multi‐center studies. In fact, following the suppression therapy, each center applied different stimulation protocols according to each unit's routine procedures and patient personalized care. Such an issue could affect the overall findings of the meta‐analysis.

Although the differences in study durations increased heterogeneity, the longer length should theoretically increase the sensitivity of pregnancy rate detection, perhaps resulting in higher accuracy. In addition, for certain outcomes, broad confidence intervals were retrieved. Such a characteristic, especially in the case of PLR, could contribute to imprecision, due to the limited number of events and reduced sample sizes of part of the included trials, as no marked sources of heterogeneity or inconsistencies were noted in both overall and subgroup analyses.

The study's ability to generalize its findings over a broad spectrum of geographic locations is another one of its many strengths. However, it is important to recognize that these regional differences could introduce variability in clinical outcomes, due to differences in healthcare systems, access to ART, and cultural or genetic factors. For instance, studies conducted in Europe and North America benefit from advanced healthcare infrastructure and widespread access to ART, whereas those from Egypt or China may face resource limitations that affect treatment availability and outcomes.^32^

Nonetheless, our study's major strength is the quality of the research we chose, as only RCTs with a minimal risk of bias overall were included in the quantitative analysis. No open trials, quasi‐randomized trials, or observational studies were included.

According to our analysis, a variety of approaches can be used before ART in women with endometriosis. However, the absence of a definitive superior technique is a call for further study.

## 5 CONCLUSIONS

A pre‐treatment with GnRH agonists for 1 or 3 months or the administration of progestins did not improve fertility rates in women with endometriosis undergoing ART. According to this network meta‐analysis, while CPR seemed similar among the different regimens, according to SUCRA analysis, a no‐treatment approach was still the best ranked method for achieving higher LBR.

However, available studies are heterogenous in terms of inclusion criteria, patient or operator blinding, and sample sizes, emphasizing the need for further high‐quality trials to confirm the available evidence.

## AUTHOR CONTRIBUTIONS

GR and PDF designed the study and wrote the manuscript; RMC and MGV searched the literature, extracted data, and revised the manuscript; GR performed statistical analyses; LDC, ASL, AE, LC, and MT critically revised the manuscript; PDF interpreted the data and drafted the manuscript. All authors read and approved the final manuscript.

## CONFLICT OF INTEREST STATEMENT

The authors have no conflicts of interest.

## Supporting information


Appendix S1.

## Data Availability

The data underlying this article are available in the article and in its Supporting Information.
