# Supplementary material for: Efficacy of hormone pre‐treatment before ART to improve reproductive outcomes in infertile women with endometriosis: Network meta‐analysis of randomized controlled trials
Source: Int J Gynaecol Obstet. 2025 Apr 12;170(3):1001–13. doi: 10.1002/ijgo.70134 (PMC12374020; doi:10.1002/ijgo.70134)
Supplement: Supplementary file 1 — Appendix S1. [file IJGO-170-1001-s001.zip › 10 - Table S2.docx]

**Table S2.** SIDE-analysis for study outcomes.

Clinical pregnancy rate

Side Direct Indirect Difference

Coef. Std. Err. Coef. Std. Err. Coef. Std. Err. P>|z|

A C -.3043388 .6204618 .6602518 1.028627 -.9645906 1.200295 0.422

A D .2166357 .5326992 -.7479475 1.076691 .9645832 1.200289 0.422

B D * .1846289 .4810252 .049132 760.6981 .1354969 760.6982 1.000

C D -.4436168 .8799477 .5209744 .8163348 -.9645912 1.200296 0.422

Live birth rate

Side Direct Indirect Difference

Coef. Std. Err. Coef. Std. Err. Coef. Std. Err. P>|z|

A C . . . . . . .

A D * -.4418328 .9331998 -.3159431 705.628 -.1258896 705.6286 1.000

B D * -.5900609 .732919 -.8543041 1396.489 .2642431 1396.489 1.000

Pregnancy loss rate

Side Direct Indirect Difference

Coef. Std. Err. Coef. Std. Err. Coef. Std. Err. P>|z|

A C .3547678 .470307 -2.679876 1.843711 3.034644 1.90275 0.111

A D -.8109302 .931695 2.223707 1.659033 -3.034637 1.902747 0.111

B D * .4700036 .6937218 -.2235976 1691.258 .6936013 1691.258 1.000

C D 1.868949 1.59098 -1.165697 1.043668 3.034646 1.902751 0.111

Implantation rate

Side Direct Indirect Difference

Coef. Std. Err. Coef. Std. Err. Coef. Std. Err. P>|z|

A B* . . . . . . .

A C* . . . . . . .

*No detectable source of inconsistency
