# Supplementary material for: Efficacy of hormone pre‐treatment before ART to improve reproductive outcomes in infertile women with endometriosis: Network meta‐analysis of randomized controlled trials
Source: Int J Gynaecol Obstet. 2025 Apr 12;170(3):1001–13. doi: 10.1002/ijgo.70134 (PMC12374020; doi:10.1002/ijgo.70134)
Supplement: Supplementary file 1 — Appendix S1. [file IJGO-170-1001-s001.zip › 9 - Table_S1_Search_Queries.docx]

**MEDLINE** (accessed through PUBMED) 83 (DUPLI)

(("ovulation induction"[MeSH Terms] OR ("ovulation"[All Fields] AND "induction"[All Fields]) OR "ovulation induction"[All Fields] OR ("ovarian"[All Fields] AND "stimulation"[All Fields]) OR "ovarian stimulation"[All Fields] OR (("assistances"[All Fields] OR "assistant s"[All Fields] OR "assistants"[All Fields] OR "assisted"[All Fields] OR "assisting"[All Fields] OR "assistive"[All Fields] OR "dental assistants"[MeSH Terms] OR ("dental"[All Fields] AND "assistants"[All Fields]) OR "dental assistants"[All Fields] OR "assistant"[All Fields] OR "helping behavior"[MeSH Terms] OR ("helping"[All Fields] AND "behavior"[All Fields]) OR "helping behavior"[All Fields] OR "assist"[All Fields] OR "assistance"[All Fields] OR "assists"[All Fields]) AND ("reproductive techniques"[MeSH Terms] OR ("reproductive"[All Fields] AND "techniques"[All Fields]) OR "reproductive techniques"[All Fields] OR ("reproduction"[All Fields] AND "technique"[All Fields]) OR "reproduction technique"[All Fields])) OR ("in vitro fertilisation"[All Fields] OR "fertilization in vitro"[MeSH Terms] OR ("fertilization"[All Fields] AND "vitro"[All Fields]) OR "fertilization in vitro"[All Fields] OR ("vitro"[All Fields] AND "fertilization"[All Fields]) OR "in vitro fertilization"[All Fields])) AND ("endometriosis"[MeSH Terms] OR "endometriosis"[All Fields] OR "endometrioses"[All Fields])) AND (randomizedcontrolledtrial[Filter])

**EMBASE**: 721

('ovarian stimulation'/exp OR 'ovarian stimulation' OR (ovarian AND ('stimulation'/exp OR stimulation)) OR 'assisted reproduction technique'/exp OR 'assisted reproduction technique' OR (assisted AND ('reproduction'/exp OR reproduction) AND ('technique'/exp OR technique)) OR 'in vitro fertilization'/exp OR 'in vitro fertilization' OR (in AND vitro AND ('fertilization'/exp OR fertilization))) AND ('endometriosis'/exp OR endometriosis) AND ('clinical trial'/de OR 'controlled clinical trial'/de OR 'randomized controlled trial'/de OR 'randomized controlled trial topic'/de)

**COCHRANE at CENTRAL**

(ovarian stimulation OR assisted reproduction technique OR in vitro fertilization) AND (endometriosis):ti,ab,kw AND ("randomized controlled trial"):pt

**CINAHL / PsycINFO / AMED / PsycExtra (accessed through EBSCO – IDEM for Italian Universities)**

(ovarian stimulation OR assisted reproduction technique OR in vitro fertilization) AND (endometriosis) Prove controllate randomizzate AND Cerca anche nel testo completo degli articoli; Applica argomenti equivalenti

**LILACS**

(ovarian stimulation OR assisted reproduction technique OR in vitro fertilization) AND (endometriosis)

**Scielo.br**

(ovarian stimulation OR assisted reproduction technique OR in vitro fertilization) AND (endometriosis)

**Clinicaltrials.gov / ICTRP (accessed through CENTRAL)**

(ovarian stimulation OR assisted reproduction technique OR in vitro fertilization) AND (endometriosis):ti,ab,kw AND ("randomized controlled trial"):pt
