# Supplementary figures and images for: Efficacy of hormone pre‐treatment before ART to improve reproductive outcomes in infertile women with endometriosis: Network meta‐analysis of randomized controlled trials
Source: Int J Gynaecol Obstet. 2025 Apr 12;170(3):1001–13. doi: 10.1002/ijgo.70134 (PMC12374020; doi:10.1002/ijgo.70134)

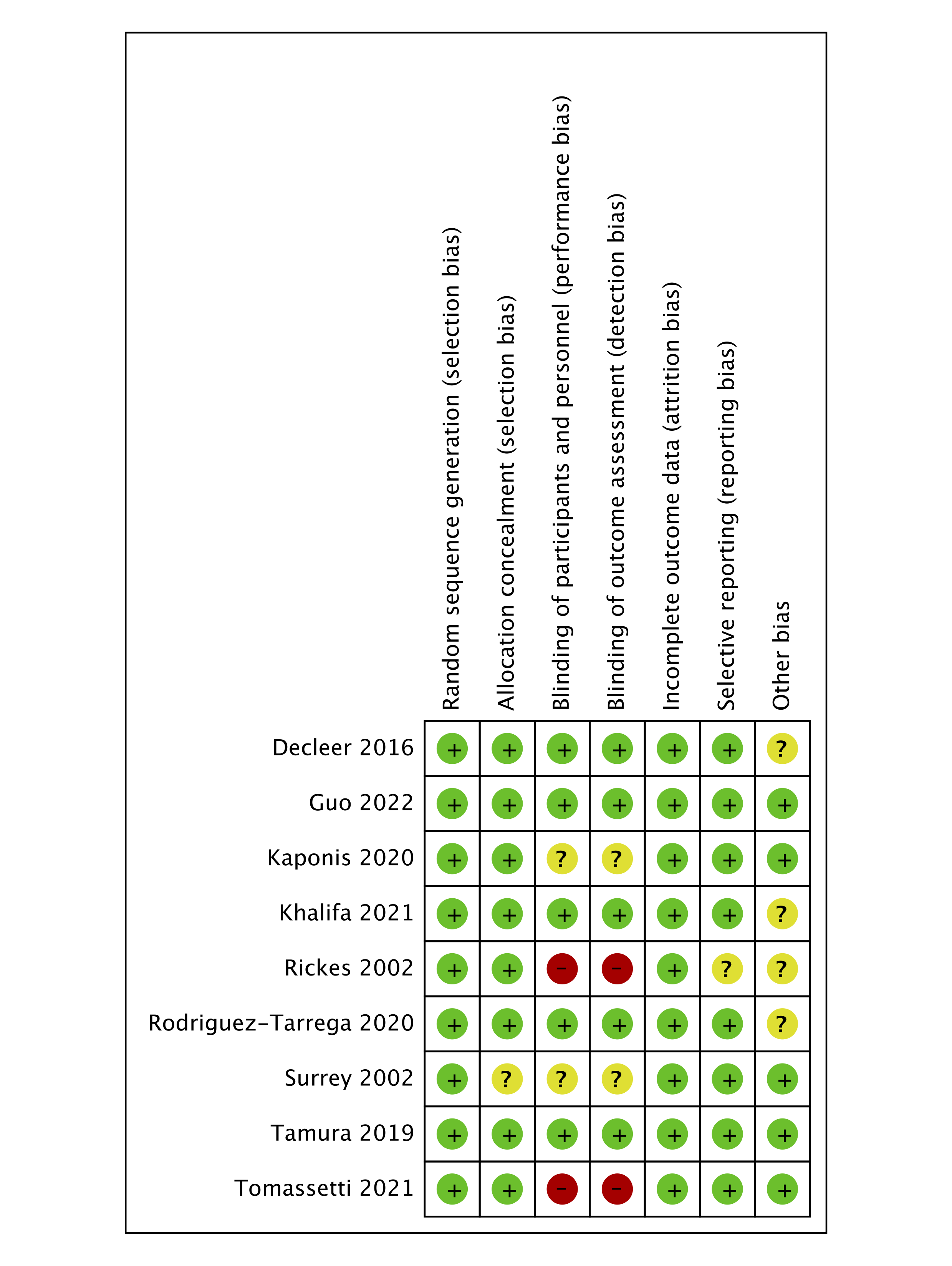

Supplement: Supplementary file 1 — Appendix S1. [file IJGO-170-1001-s001.zip › 11 - Figure_S1a_Risk of bias summary.tiff]

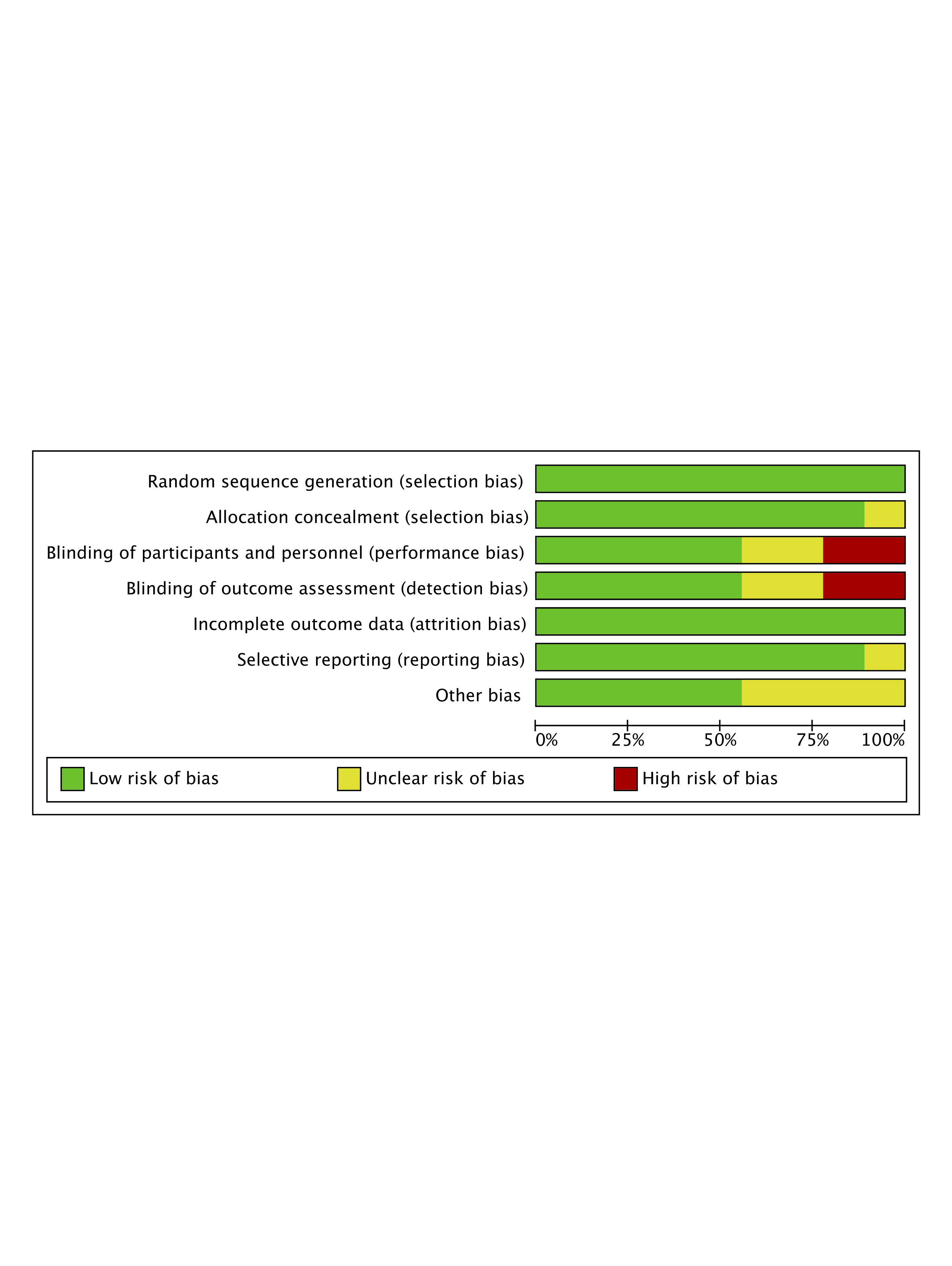

Supplement: Supplementary file 1 — Appendix S1. [file IJGO-170-1001-s001.zip › 12 - Figure_S1b_Risk of bias graph.tiff]

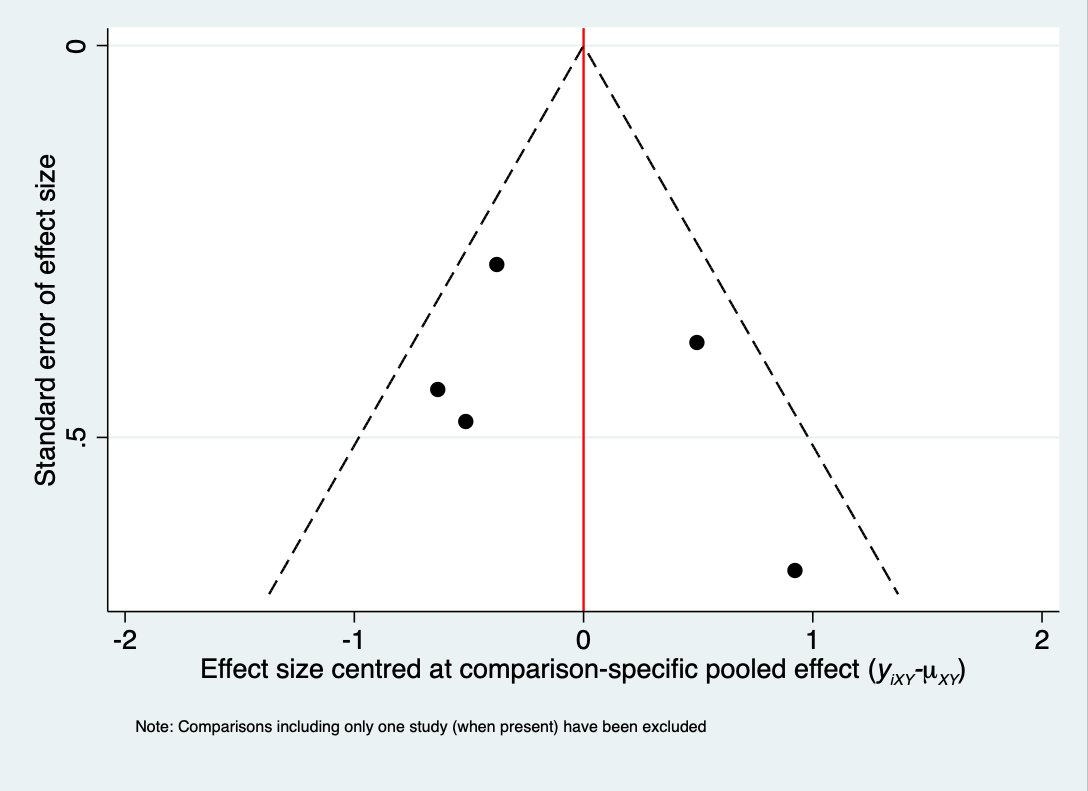

Supplement: Supplementary file 1 — Appendix S1. [file IJGO-170-1001-s001.zip › 13 - Figure_S2_funnel.tif]

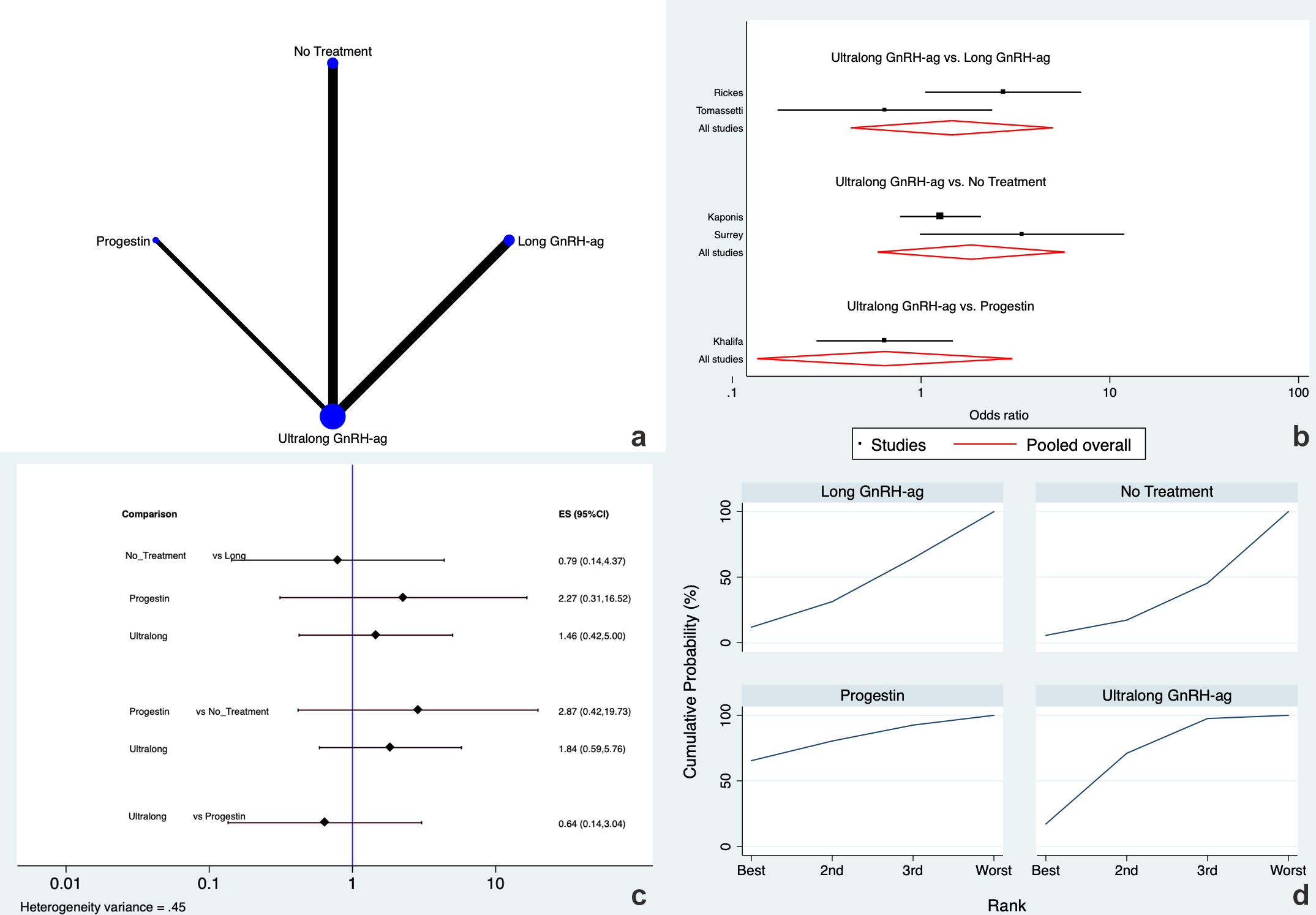

Supplement: Supplementary file 1 — Appendix S1. [file IJGO-170-1001-s001.zip › Figure_S10.tif]

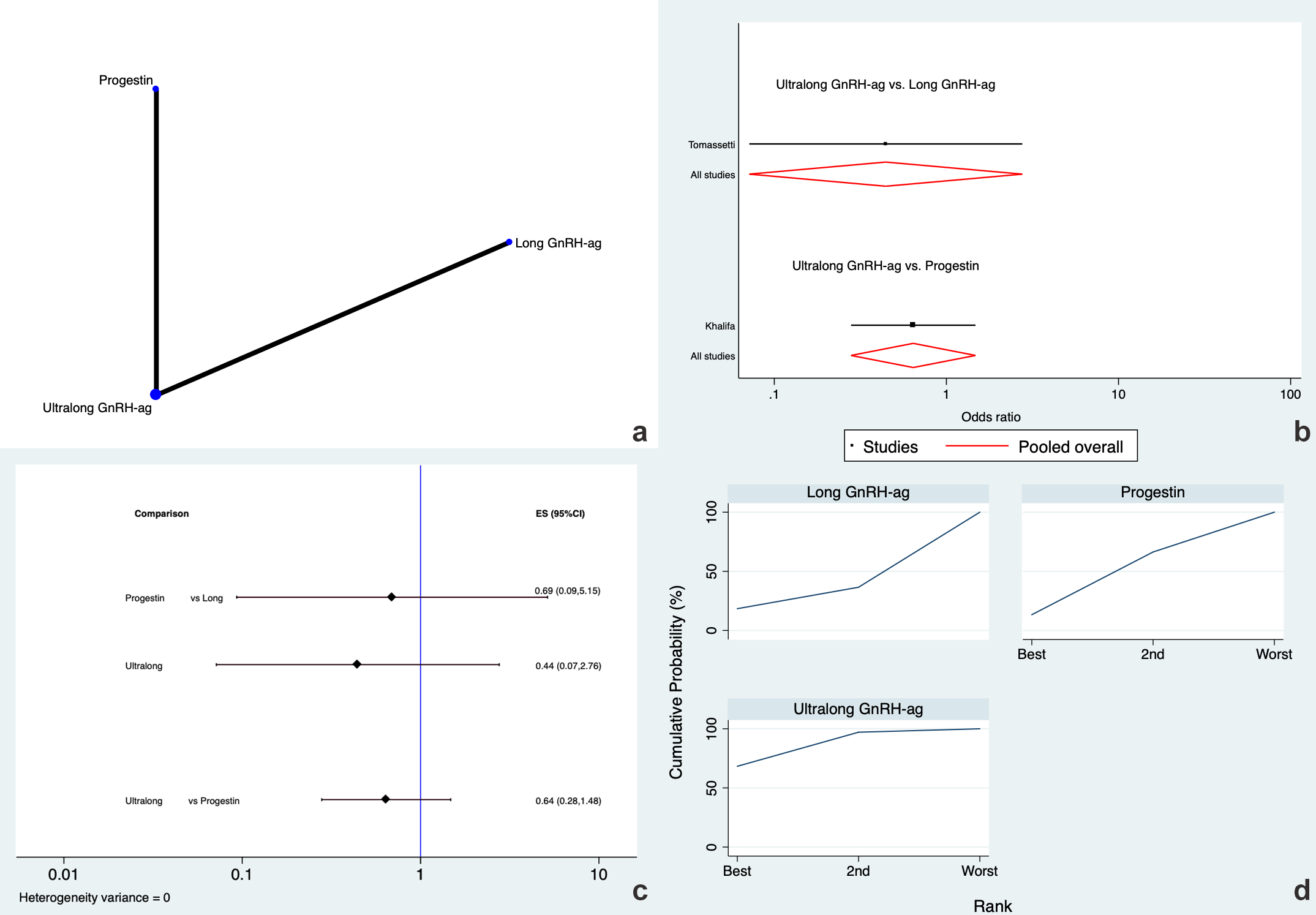

Supplement: Supplementary file 1 — Appendix S1. [file IJGO-170-1001-s001.zip › Figure_S11.tif]

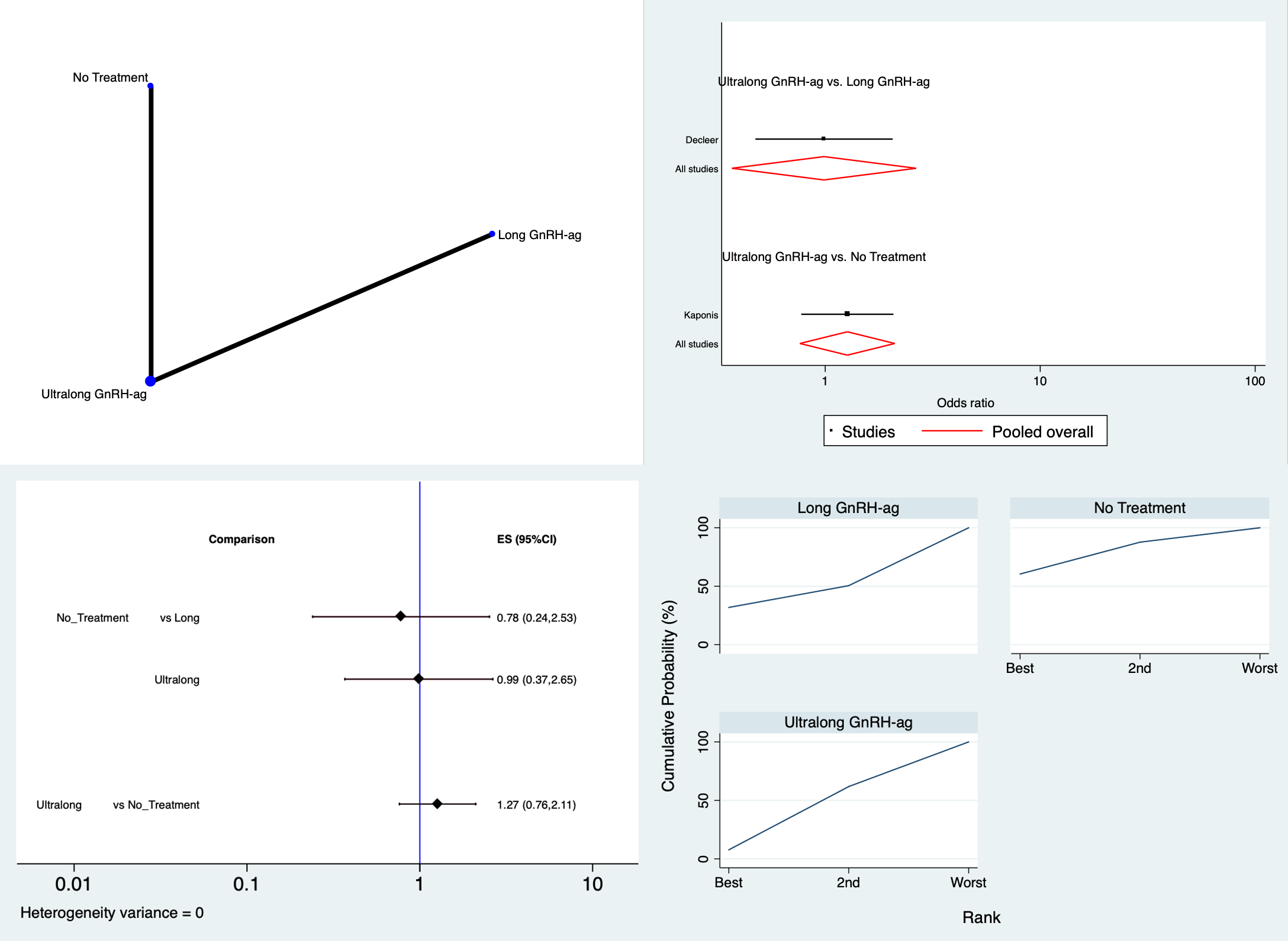

Supplement: Supplementary file 1 — Appendix S1. [file IJGO-170-1001-s001.zip › Figure_S3.tif]

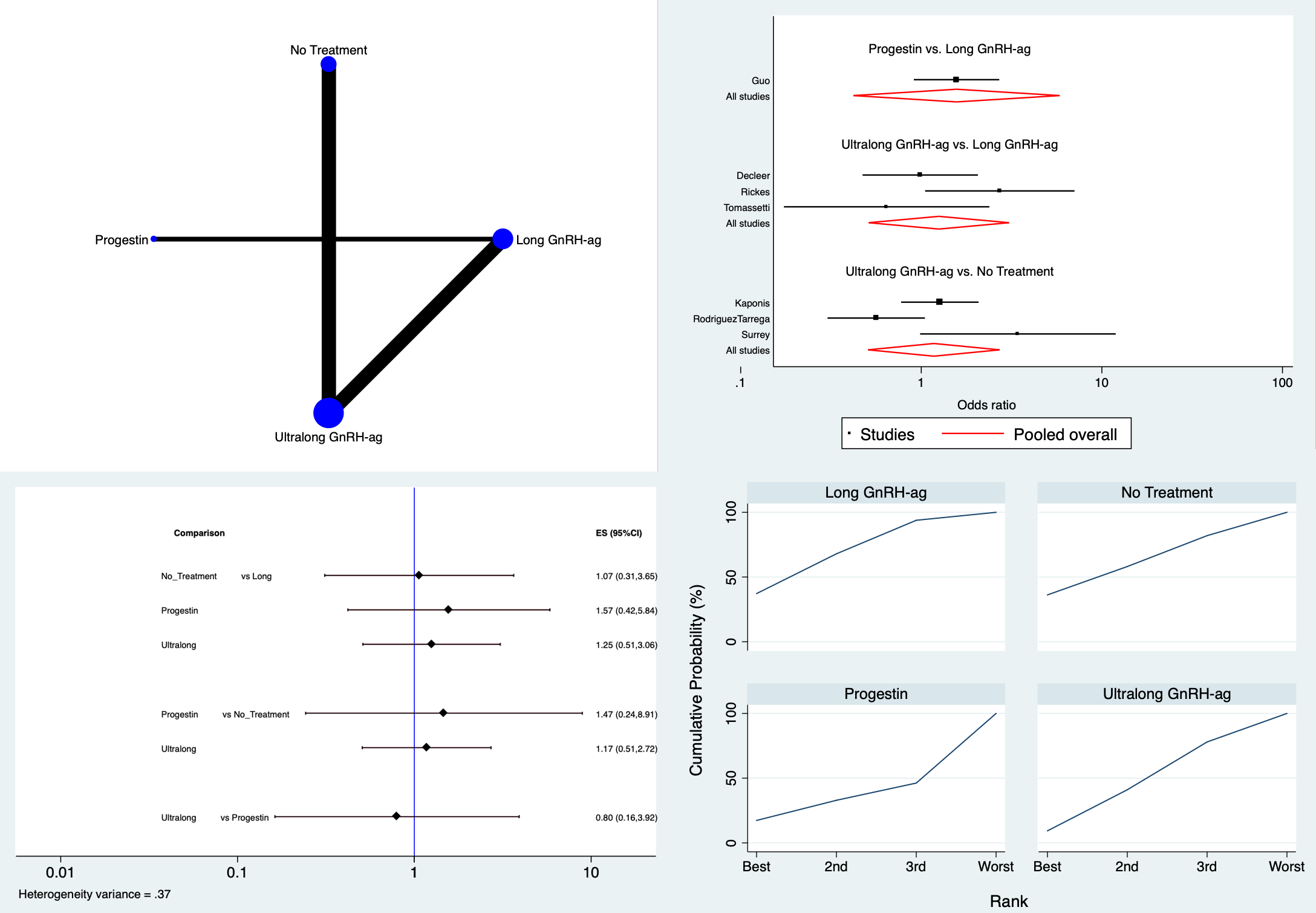

Supplement: Supplementary file 1 — Appendix S1. [file IJGO-170-1001-s001.zip › Figure_S4.tif]

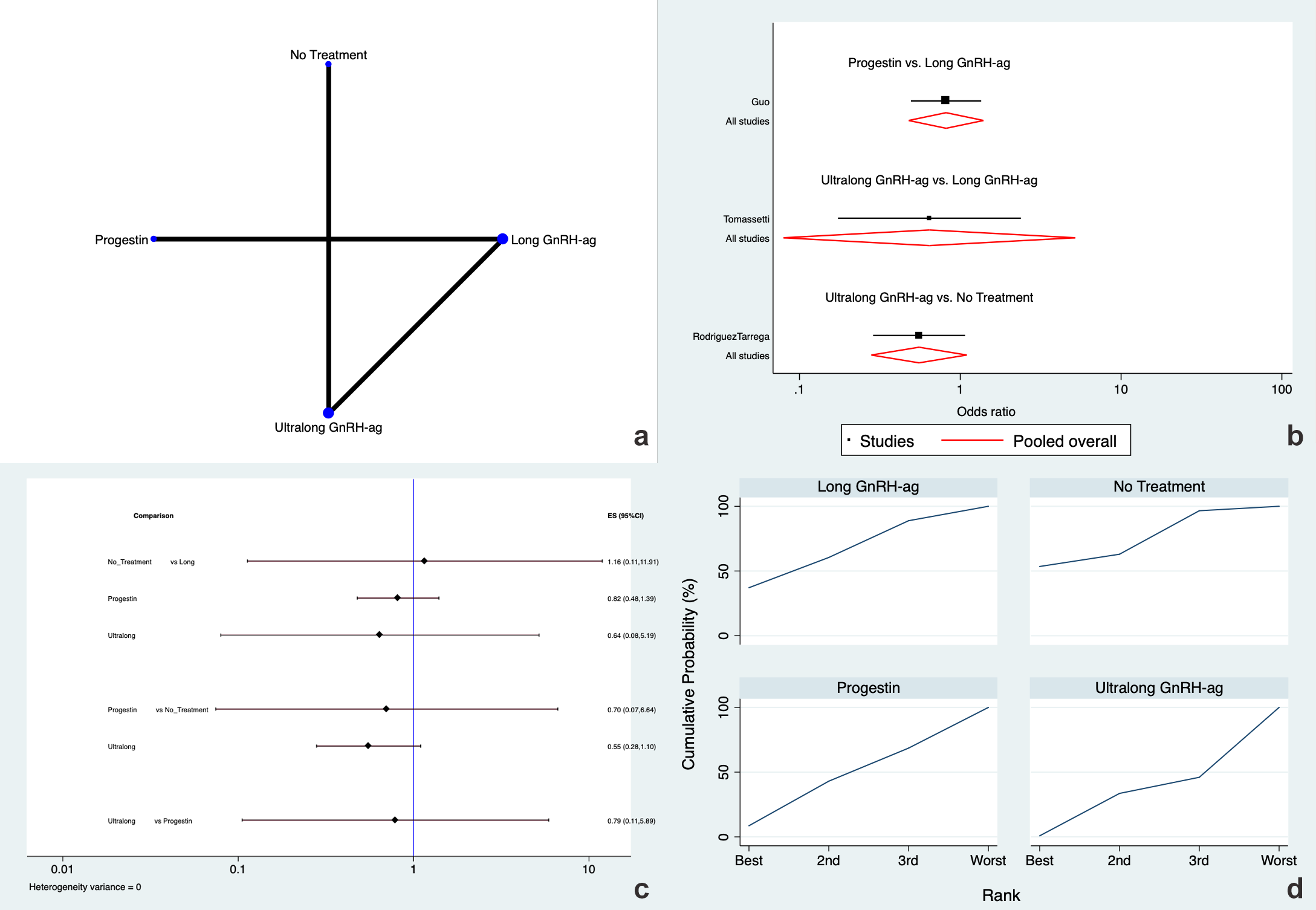

Supplement: Supplementary file 1 — Appendix S1. [file IJGO-170-1001-s001.zip › Figure_S5.tif]

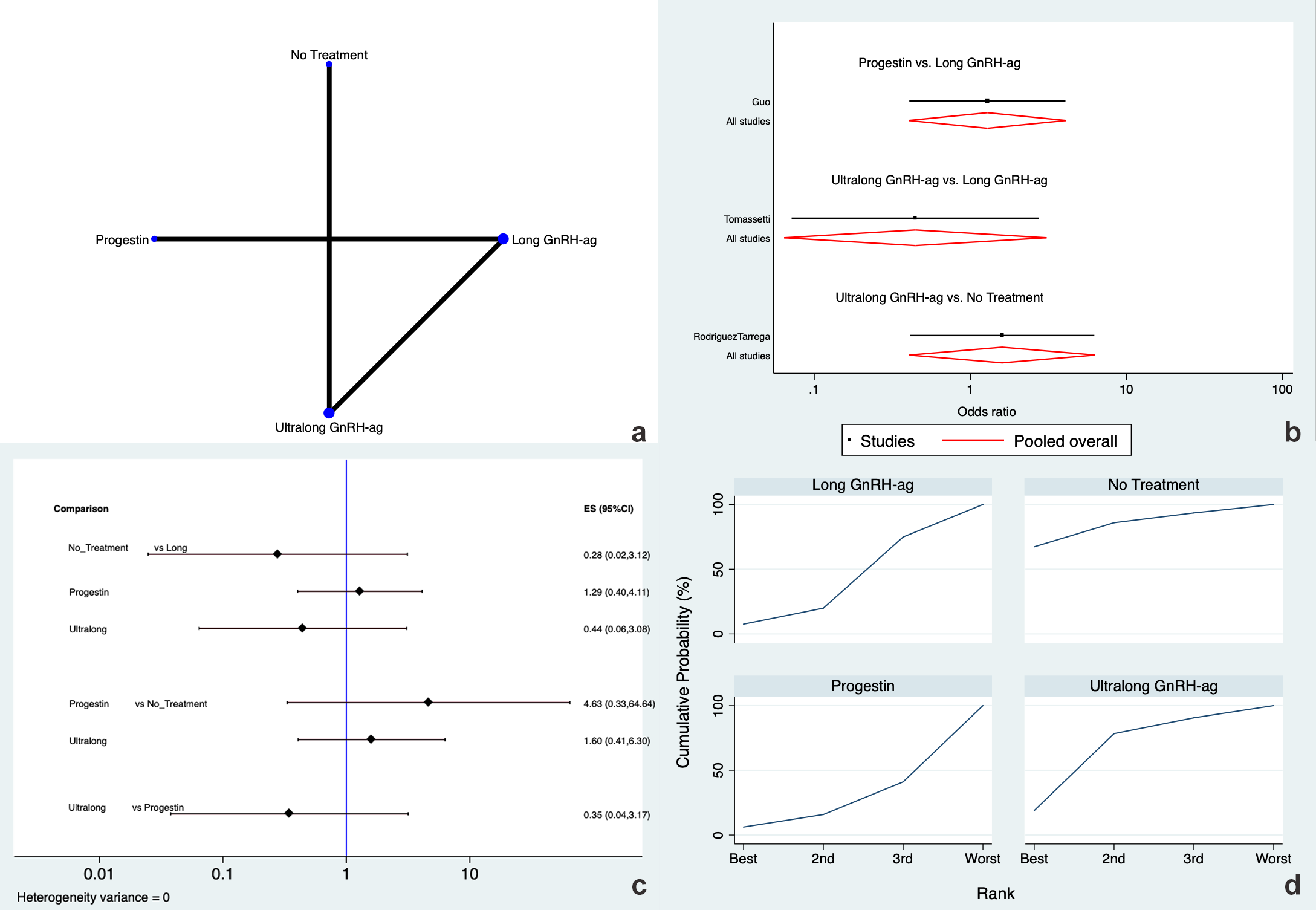

Supplement: Supplementary file 1 — Appendix S1. [file IJGO-170-1001-s001.zip › Figure_S6.tif]

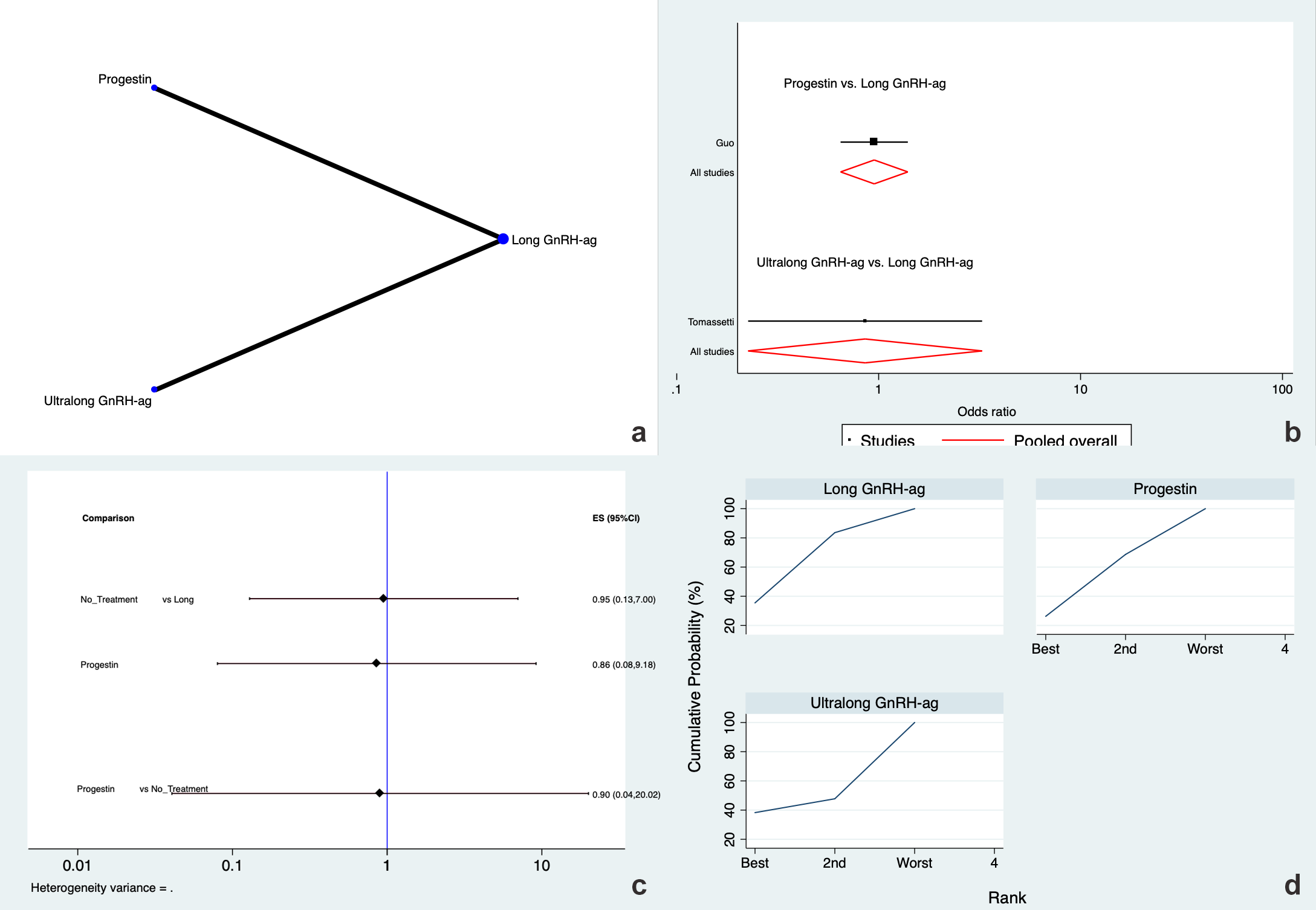

Supplement: Supplementary file 1 — Appendix S1. [file IJGO-170-1001-s001.zip › Figure_S7.tif]

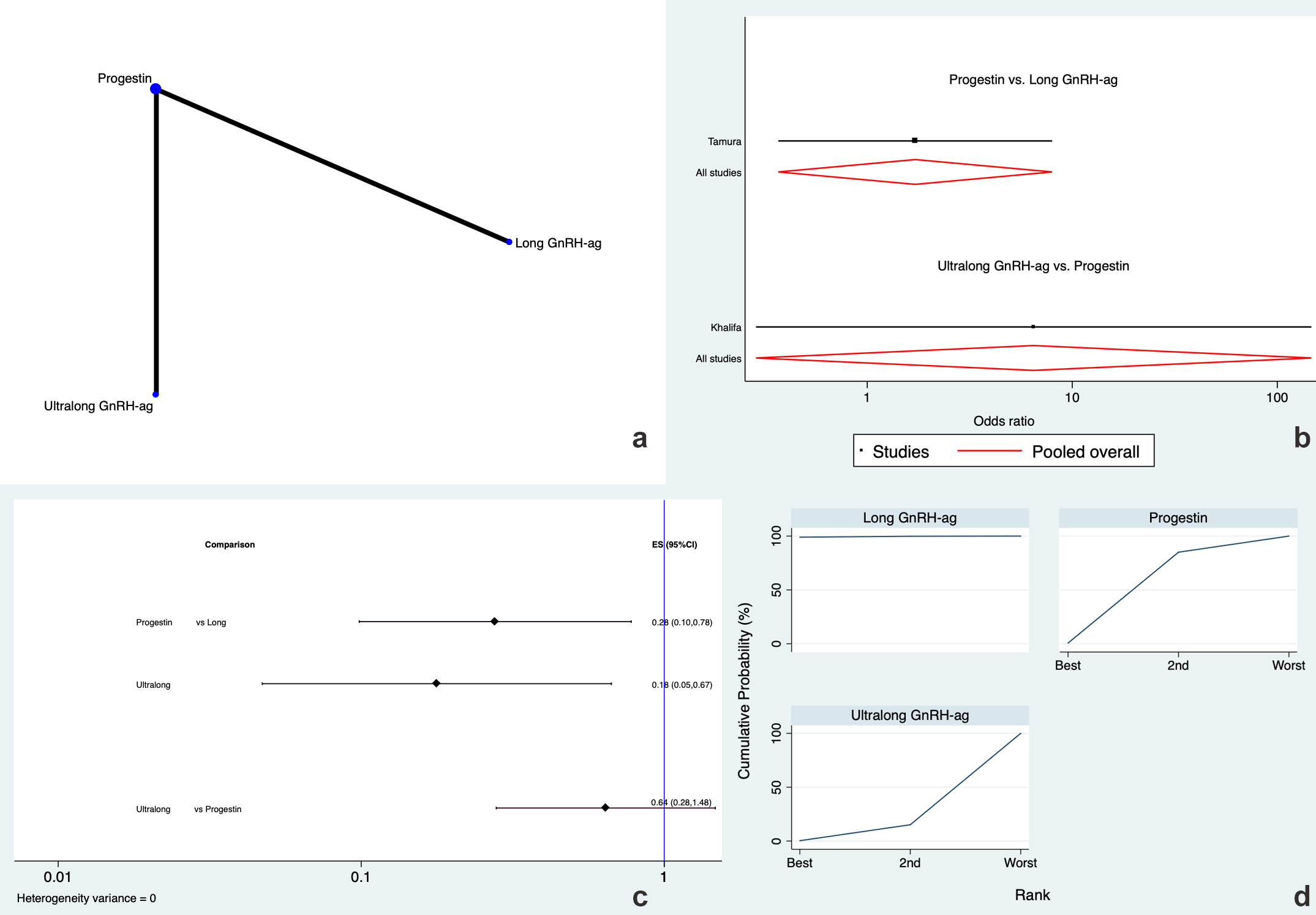

Supplement: Supplementary file 1 — Appendix S1. [file IJGO-170-1001-s001.zip › Figure_S8.tif]

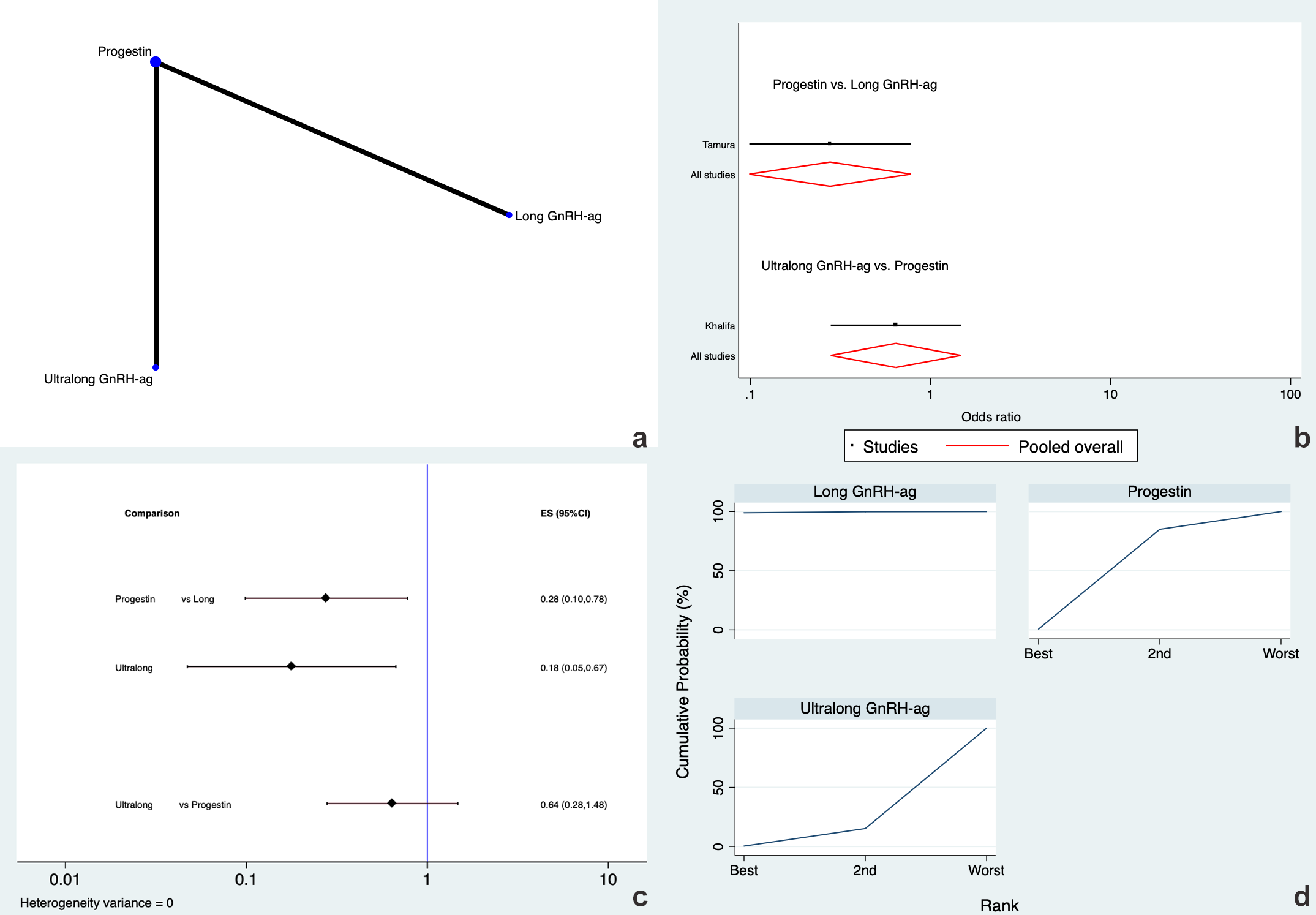

Supplement: Supplementary file 1 — Appendix S1. [file IJGO-170-1001-s001.zip › Figure_S9.tif]
